# Adverse Drug Reactions in a Complementary Medicine Hospital: A Prospective, Intensified Surveillance Study

**DOI:** 10.1155/2012/320760

**Published:** 2012-01-17

**Authors:** M. Süsskind, P. A. Thürmann, C. Lüke, E. Jeschke, M. Tabali, H. Matthes, T. Ostermann

**Affiliations:** ^1^Research Institute Havelhöhe, Gemeinschaftskrankenhaus Havelhöhe, 14089 Berlin, Germany; ^2^Philipp-Klee Institute of Clinical Pharmacology, HELIOS KlinikumWuppertal, Witten/Herdecke University, 42283 Wuppertal, Germany; ^3^Center for Integrative Medicine, Witten/Herdecke University, 58313 Herdecke, Germany

## Abstract

*Background*. Anthroposophic medicine is one of the widely used approaches of complementary and alternative medicine. However, few prospective studies have generated safety data on its use. *Objectives*. We aimed to assess adverse drug reactions (ADRs) caused by anthroposophical medicines (AMEDs) in the anthroposophical Community Hospital Havelhoehe, GERMANY. Study Design and Methods. Between May and November 2007, patients of six medical wards were prospectively assessed for ADRs. Suspected ADRs occurring during hospitalization were documented and classified in terms of organ manifestation (WHO SOC-code), causality (according to the Uppsala Monitoring Centre WHO criteria), and severity. Only those ADRs with a severity of grade 2 and higher according to the CTCAE classification system are described here. Results. Of the 3,813 patients hospitalized, 174 patients (4.6%) experienced 211 ADRs (CTCAE grade 2/3 *n* = 191, 90.5%, CTCAE grade 4/5 *n* = 20, 9.5%) of which 57 ADRs (27.0%) were serious. The median age of patients with ADRs (62.1% females) was 72.0 (IQR: 61.0; 80.0). Six patients (0.2%) experienced six ADRs (2.8% of ADRs) caused by eight suspected AMEDs, all of which were mild reactions (grade 2). *Conclusion*. Our data show that ADRs caused by AMEDs occur rarely and are limited to mild symptoms.

## 1. Introduction

The term “complementary and alternative medicine” (CAM) describes a variety of approaches to medical theory and practice including homeopathy, herbal medicine, naturopathy, anthroposophic medicine, and traditional Chinese medicine, all of which are becoming increasingly accepted by practitioners of conventional medicine [1, 2]. One of the main reasons for such a high level of interest and demand for complementary medicine is the belief of patients and physicians that “natural” products or services are safer than conventional allopathic products [3]. Although there is substantial evidence that herbal medicines can cause serious adverse reactions [4], there is still a lack of knowledge on the occurrence of adverse reactions to CAM [5].

Anthroposophic medicine is one of the most frequently used traditional European approaches of CAM which involves the use of a variety of therapies, including art, music, eurythmy (movement therapy), massage, and counselling as well as anthroposophic medicines (AMEDs) [6, 7]. Anthroposophical pharmacotherapy uses substances from the mineral, plant, and animal kingdoms [8, 9]. Substances undergo a variety of pharmaceutical processes and are applied in many different dilutions like in homeopathy, but also in different preparations and combinations. Apart from drug intake, AMEDs may also be applied as external treatments with oils, ointments, and plant extracts.

Only few prospective studies have generated safety data on AMEDs and most of them result from outpatient investigations. In a prospective cohort study in 662 patients using AMED chronically only 20 patients reported possible or probable ADRs associated with AMED, none of them was serious [10, 11]. In particular, no data have been reported so far on AMED-associated ADRs occurring in hospitals.

In contrast, the nature and frequency of ADRs following the use of conventional drugs in hospitalized patients have been thoroughly studied in recent years [12–14]. Thus, comprehensive ADR surveillance in a hospital setting promises to be an efficient tool to detect all kind of ADRs and all grades of severity.

The aim of this prospective pharmocoepidemiological study was to estimate the incidence of adverse drug reactions (ADRs) with a special focus on ADRs caused by CAM and, in particular, AMEDs. We therefore conducted this study in a community hospital, where AMEDs are frequently used along with conventional drugs and therapies. In order to provide for a valid basis of our detection method and to prove comprehensiveness, ADRs associated with the use of allopathic pharmaceuticals used were also collected and can be compared with data published in the international literature [15–17].

## 2. Material and Methods

### 2.1. Patients

A prospective, comprehensive collection of ADRs on six medical wards, including two gastroenterology wards, two general internal medicine wards, one pneumology ward, and one cardiology ward, was carried out at the Community Hospital Havelhöhe (GKH) in Berlin over a six-month period between May 15 and November 15, 2007. The GKH is a community hospital with a focus on anthroposophic medicine where about 63% of inpatients on medical wards receive one or more CAM drugs (median 3 (IQR: 1; 5)), of which 92% receive at least one AMED (median 3 (IQR: 1; 5); personal communication), that is, approximately 58% of all patients admitted receive an AMED.

Almost half of patients (46%) in this study came from the geographical catchment area, 32.9% of patients admitted came from further regions within Berlin (approx. 30 km), and 21.1% were admitted from other regions in Germany or from abroad. The latter came to the GKH with the intention of receiving anthroposophic medicine.

### 2.2. Anthroposophic Medications (AMEDs) Used in Hospital

For this study, we used data from the hospital pharmacy to describe usage of the most frequently prescribed substances during the study period on the wards under surveillance, regardless of dilution, pharmaceutical processing, or combination with other substances (Table 1). For example, the frequently prescribed plant *Arnica montana* is included in 19 different preparations of AMEDs (i.e., whole plant and root preparations in 4 different dilutions, preparations of *Arnica* processed with four other plants, essence, gel, ointment, and oil in combination with cuprum), which are administered orally, subcutaneously, or externally.

### 2.3. Comprehensive ADR Surveillance and Data Collection

Prior to investigation, the physicians and nurses of all participating wards were trained in order to increase awareness for ADRs according to a program developed for an educational intervention to improve primary care physicians ADR reporting [18]. Besides principles and theories of ADRs, typical ADR trigger symptoms and case studies were discussed. Clinical staff was invited to refer directly or by phone to the investigator to report potential ADRs.

A specially trained physician conducted the investigation. She visited the wards three times a week, questioned attending physicians and nurses, and examined the patients' drug charts and medical records for evidence of potential ADRs. A set of trigger symptoms used by the German Network of Pharmacovigilance Centers [19] was applied as were changes in medication and predefined laboratory parameters surpassing specified threshold values (according to CTCAE criteria more than grade II) [20], for detection of ADRs. Nurses' notes were screened for subjective symptoms, such as nausea, vomiting, or headache. In case of suspected ADRs, the investigator conferred with the attending physicians and nurses.

Patient data collected included age, gender, weight, height, date of admission and discharge, Kanofsky index (if applicable), all diagnoses (ICD-10), and drugs (dose, route, and duration of administration). ADR description included start, duration and outcome, suspected drug(s), and management strategies (e.g., drug withdrawal, dose reduction, and additional treatment).

Patients with ADRs obviously resulting in hospital admission and chemotherapy-induced ADRs (such as nausea, vomiting, and leucopenia) were excluded in the study protocol. Recurrences of clostridium-associated diarrhea were subsumed as one ADR.

### 2.4. Assessment and Classification of ADRs

ADRs were classified according to the definition of Edwards and Aronson [21]. We restricted ADR classification to type A, pharmacologic effects which are dose dependent and predictable, and type B, effects which are dose independent and unpredictable, which are the most frequent and accessible types of ADRs recognized in the literature. CIOMS criteria were applied for case definitions [22]. As sources of information for the verification of potential adverse effects, the manufacturer's summary of product characteristics [23], Lexi-Comp Online [24], a Handbook of ADRs [25], and Medline search were used.

Suspected ADRs were assessed and defined according to internationally accepted criteria [22] and classified in terms of organ manifestation (WHO SOC-code), causality [26, 27], and severity [20]. Causality was defined and assessed according to definitions of the Uppsala Monitoring Centre and was classified as certain, probable/likely, possible, unlikely, conditional/unclassified, or unassessable/unclassifiable. Only “possible,” “probable,” and “definite” ADRs were taken into consideration; such were also classified in terms of causality according to Naranjo et al. [26]. Furthermore, ADRs were classified according to the International Conference on Harmonization criteria as serious and nonserious, as well as in terms of severity according to the Common Terminology Criteria for Adverse Events (CTCAE) in grades 1 to 5 (mild-letal) [20, 26, 28, 29].

Assessment for causality and severity was done independently by at least two investigators. Internal quality assurance proceedings were arranged in regular meetings with the clinical pharmacologist. In addition, if any discrepancies in scoring were discussed between the investigators in conjunction with the clinical pharmacologist, specific medical questions were reconsidered with the respective medical specialist. The consent attained this way was accepted as the final ADR classification.

### 2.5. Mistletoe-Induced ADRs

Although mistletoe (*Viscum album*) can be considered a herbal chemotherapeutic agent due to its cytotoxic properties, we decided to include mistletoe-associated ADRs although ADRs following allopathic cytotoxic drugs had been excluded. Other characteristics, such as modulation of the immune system, have been shown for mistletoe [30–33]. As a consequence, mistletoe is prescribed as a remedy for diverse diseases, for example, rheumatological diseases, hepatitis, and cancer and can be coded using three different ATC codes (L01CP01 plant cytostatics, M09AP06 plant preparations for musculoskeletal disorders, and C02KP02 plant antihypertensive).

Due to its immunomodulating properties mistletoe provokes local (reddening and swelling) and systemic (rise in temperature/fever) reactions, which can be considered type A reactions according to Rawlins [34].

Reactions exceeding the expected measure were classified separately for intravenous or local application and documented in a special section in the database. The criteria are listed below and follow CTCAE criteria: increase of body temperature axillary >38.0°C (CTCAE II°, hypersensitivity including drug reaction); reddening at the assured injection site >50 mm (CTCAE I); itching exceeding the injection site (CTCAE II, pruritus/itching); local swelling >10 mm (CTCAE II°II); swelling of the lymph node >10 mm; unexpected, uncharacteristic reaction.

### 2.6. Collection and Documentation of Drug Prescriptions

Data on medicines prescribed in the hospital were collected and recorded manually. In order to achieve a high completeness of data, the recording of all drugs, including causative drugs, was performed only for patients with an ADR and not for patients without a suspected ADR.

ADR-associated drugs were classified by the anatomical therapeutic chemical (ATC) code.

### 2.7. Data Analysis

Collected data were structured hierarchically, allowing for evaluation of a patient and the admission and ADR level using the database QuaDosta, a PostgreSQL database [35]. All statistical analyses were performed using SPSS 16.0 for Windows. Descriptive analysis was used to determine patients with ADRs and ADR frequencies according to SOC code, ATC code, and severity. Medians and interquartile ranges (IQRs) were calculated for continuous, not normally distributed data. The two-tailed chi-square test was used to analyze differences in proportions and the median test was used to analyze age differences. A *P-*value of less than 0.05 was regarded as indicating a statistically significant difference. Subgroup analyses were performed for drug groups according to ATC code.

## 3. Results

### 3.1. Patient Characteristics

Over a six-month period, a total of 3,813 patients (2,063 females; 54.1%) with a mean age of  67 ± 10  years, median 55 years, were admitted, stayed at least one night, and were assessed for ADRs (Table 2).

The leading admittance diagnoses were coronary heart disease (5.9%), fatigue (3.5%), dyspnoea (2.7%), pain (2.7%), pneumonia (2.2%), and lung cancer (2.2%).

12.9% of the patients had different types of cancer, 3.5% received chemotherapy, and 9.1% received mistletoe during the hospital stay.

174 patients (4.6%) experienced at least one ADR during their stay in hospital (ADR). The ADR-experiencing patients were more likely to be female (62.1% females, *P* < 0.001) and older when compared to patients without ADR (median age 72 years, IQR 61.0; 80.0, see Table 2, *P* < 0.001).

### 3.2. ADRs

A total of 211 ADRs (including the 6 ADRs caused by CAM) were detected and documented on the basis of WHO/UMC criteria. They were judged as probable in 123 cases (59%), 72 were judged as likely (34%), and 10 as definite (5%). This assessment was in good accordance with the Naranjo scoring, where 105 ADRs were considered probable (49.8%), 99 possible (46.9%), and seven as highly probable (3.3%).The greater number of ADRs with *n* = 184 (84.4%) were identified as type A reactions.

Distribution of severity and seriousness of ADR is shown in Table 3. The majority of ADRs were of severity grades II and III and 26.1% were deemed serious according to [36] (see Table 3). Of these 55 serious ADRs, 33 led to a prolongation of the hospital stay and 20 were life threatening or potentially life threatening. Eleven patients with serious ADRs did not fully recover, two patients died: one from acute renal failure (vancomycin and gentamicin) and one from Salmonella sepsis (prednisolone).

All 156 nonserious ADRs abated without sequelae and the outcome of four patients was unknown.

The organ systems mainly affected were the gastrointestinal tract (*n* = 110, 52%; main symptoms: diarrhea *n* = 23, constipation *n* = 22, nausea *n* = 18, GI-bleeding *n* = 8, and gastric ulcer *n* = 6), the CNS (*n* = 35, 17%; main symptoms: vertigo *n* = 7, somnolence *n* = 5, and headache *n* = 3), and psychiatric disorders (*n* = 24, 11.4%; main symptoms: psychosis *n* = 3, disorientation *n* = 2, and aggressiveness *n* = 2). The most frequent adverse effects overall were diarrhea, constipation, nausea, and antibiotic-associated diarrhea.

### 3.3. Drugs Associated with ADRs

Altogether 389 (multiple nominations possible) drugs were associated with 211 ADRs. All drugs causing the ADRs were initiated in the hospital. The most common groups of ADR-related drugs were anti-infectives (*n* = 124, 32%) followed by opiates (*n* = 45, 12%), cardiovascular drugs (*n* = 59, 12.3%), steroids (*n* = 35, 9%), and antithrombotics (*n* = 34, 8.7%) (see Table 4).

### 3.4. ADRs Associated with AMED Medication

Altogether only six CAM-associated ADRs were detected. All associated medicaments were AMEDs. According to the CTCAE scale, all ADRs were grade II and none were serious. All patients had recovered from these ADRs at the time of discharge.  Table 5 shows details of AMED-associated ADRs. Assuming that approximately 58% of all patients admitted during the surveillance period received AMED, only 0.27% (6 of 2212 AMED-exposed patients) suffered an AMED-associated ADR.

All three mistletoe reactions (erythema and fever) occurred between 18 and 24 hours after injection, as well as the erythema after the injection of *Equisetum*, *Formica*, *Arnica,* and *Levisticum*.

The maculopapular exanthema after hepatodoron (*Fragaria vesca*/*Vitis vinifera*) affecting both forearms appeared two days after the first oral application of the drug and was considered a type IV reaction. The skin eruption appearing on the chest of the patient about 5 hours after the first oral application of *Gentiana*,* Bryophyllum,* and metamizole was judged to be a type II (cytotoxic) reaction.

## 4. Discussion

To our knowledge, this is the first prospective study on the burden of adverse drug reactions (ADRs) in a hospital, where patients are treated with standard drugs and complementary and alternative medicine (CAM). In this specific case, most patients were treated with anthroposophic medicine (AMED). In order to demonstrate the comprehensiveness of our detection method, ADRs caused by conventional drugs were also captured. In comparison with the literature, the estimation of 4.6% of all hospitalised patients with ADRs in our study is comparable to the results of a systematic review from 2002 by Wiffen and colleagues [37], who estimated an ADR incidence of 3.7% worldwide from studies undertaken after 1985. Our results also compare well with findings of Moore et al. on internal medicine wards where 6.6% of all patients suffered from ADRs [38]. However, Lazarou at al. estimated an ADR incidence of 10.9% in their meta-analysis of 39 prospective studies undertaken in American hospitals between 1966 and 1996 [15]. We excluded ADRs associated with chemotherapy, which are generally expected and also not recorded in some of the studies [37, 39]. Fattinger et al. report the ADR incidence of 11% in two Swiss hospitals, which is reduced after the exclusion of chemotherapy-induced ADRs to 8% [40].

When looking at drug classes responsible for ADRs, our results also correspond to data in the literature, where antibiotics, opioids, antithrombotics, and cardiovascular agents are the most frequently documented drugs associated with ADRs in hospitalized patients [39].

Discrepancies in results of all ADR studies are well known, mainly methodological differences have been found responsible and were thoroughly investigated and described [37, 39, 41]. Taken together, nature and frequency of the ADRs in our study compare well with data published in the literature for a comprehensive collection of ADRs in hospitals and prove the reliability of our approach.

### 4.1. ADRs Associated with the Use of AMED

Our main concern was the investigation of the burden of ADRs occurring in hospitals associated with AMED.

We detected six AMED-associated ADRs (2.8% of ADRs) in approximately 0.27% of AMED-exposed patients, all of which were of mild severity (CTCAE grade II), and none of which were serious.

In three cases, the ADR-suspected drugs were mistletoe extracts associated with three ADRs, all with a probable causality; a rise in temperature >38.5°C was recorded for two cases, whereas a local reaction with swelling and reddening in a diameter of 8 × 8 cm was observed in the other. All these reactions appeared after the first administration of the extracts to the patients. The detection of only three reactions fulfilling these criteria appears to be rather low. This is probably due to the fact that local reactions of mistletoe are an indicator of the immunomodulatory action and seen as desired reactions and therefore are not identified and documented as exceptional events or even ADRs.

Unfortunately, we cannot perform a precise incidence calculation from our data since the exact number of patients exposed is unknown. Even in the case that all 492 patients in our cohort with a diagnosis of cancer received mistletoe, 0.61% of these patients (3/492) have experienced an ADR. This calculation probably underestimates the true incidence. A comparison with the literature shows that the above-described ADRs are reported in most studies of mistletoe therapy (mostly concerning tumour patients) and are also listed in the new summary of product characteristics of the products (SPC of Helixor, Abnoba, Iscucin and Iscador). In the case of Abnoba f.ex., ADR rates between 0.9 and 47% (including local and systemic ADRs) were reported [42]. According to a clinical study of Büssing et al. 21% of patients treated with mistletoe developed an erythema >3 cm [43].

As mistletoe extract is a drug quite frequently administered in anthroposophic medicine and the most frequently prescribed complementary drug in oncology (approx. 12 mio. daily doses in 2007 in Germany) [44], this clinically important ADR should be a matter of reporting. Indeed the drug safety database of the Drug Commission of the German Medical Association (AkdÄ) includes a list of 113 reports of 349 suspected ADRs in connection with mistletoe administration up to April 2010 (personal communication). The sources of these reports include spontaneous reporting as well as systematic research. Unfortunately, causality is not uniformly assessed, and, in many cases, multiple medications were administered, thus hampering a clear correlation of ADRs and mistletoe.

There is some evidence that the local reactions and probably also the systemic ADRs (mostly elevated temperature) are dose dependent and disappear with dose reduction, whereas the individual dose causing an ADR differs widely between patients and even the same patient may react in a different way to different mistletoe preparations [45]. Almost all reported ADRs had a mild course resulting in complete recovery [42].

The three other cases of ADRs possibly associated with AMED occurred in a local reaction (erythema) following the subcutaneous injection of *Equisetum* and *Formica* and of *Arnica* and *Levisticum*. After the readjustment to oral administration of these drugs, the erythema declined. Hepatodoron (*Fragaria vesca*/*Vitis vinifera*) is one of the most frequently prescribed AMEDs in our hospital (roughly 1640 daily doses during the study period, considering the regular prescription of 4–6 tablets per day) and it is classified as a possible causative for the maculopapular exanthema. From 1990 to April 2010 two suspected ADRs from spontaneous reporting are listed for hepatodoron in the above-mentioned database of the Drug Commission of the German Medical Association.

Another ADR-suspected AMED from the list of most frequently used AMEDs was bryophyllum together with gentiana. As they were administered concomitantly with metamizole, a drug frequently associated with exanthema, the causality was classified as only possible.

In summary, all ADR-suspected AMEDs belong to the most frequently administered drugs in our hospital. Except ADRs following mistletoe injections, which are classified as probable in all cases, the causality for the other AMEDs was considered as only possible.

## 5. Limitations

The exclusion of chemotherapy-induced ADRs could have led to an underrating of ADRs caused by drugs given concomitantly to chemotherapy. This would apply in our study preferably to ADRs occurring after the frequently coadministered mistletoe. However, the symptoms caused by mistletoe, consisting mainly of local erythema at the injection site, differ strongly from the symptoms caused by chemotherapy (e.g., nausea, vertigo) and should not be missed.

Unfortunately, we were unable to document the medications of all patients comprehensively, precluding an exact estimation of incidence of ADRs per prescriptions or patients exposed. Moreover, in the case of AMEDs, neither dose nor duration of therapy can be standardized, as described (e.g., in the case of the pronounced interindividual reactions of patients to different doses and/or preparations of mistletoe). Therefore, a simplified calculation using the concept of daily drug doses (DDD) would give an unreliable and imprecise estimate of exposure.

As case definition and causality assessment of ADRs is an issue in all studies estimating ADRs, we undertook several measures to provide for a reliable assessment, that is, two independent physicians used two algorithms in parallel and in case of disagreements a third assessor and/or medical specialist was involved.

Finally, this study unlike others was not designed to assess effects or efficacy of AMEDs [46].

## 6. Conclusion

Despite the skepticism towards CAM and AMEDs [47], our data show that ADR surveillance is possible and a reasonable safety profile of AMEDS can be assumed. ADRs associated with allopathic medication detected in our study were comparable with regard to incidence and nature to published data, underlining the comprehensiveness of our prospective survey.

In summary, our results suggest that ADRs caused by AMEDs occur only with a low incidence and produce mainly mild to moderate symptoms. Since mistletoe extracts were the predominantly used AMEDs in this cohort of patients, most ADRs were associated with mistletoe extracts. This study showed the difficulties to comprehensively document the complete medications of all patients. Thus interpretation of our results must be done with great caution.

## Figures and Tables

**Table 1:** Most frequently prescribed anthroposophic medications (AMEDs) based on plant and metal preparations^a^ at the Community Hospital Havelhoehe.

Plant/metala	Main indication	Ampoules [mL]	Dilutions [mL]	Triturations [g]	Tablets [*n*]	Ointments/oils [g]
*Kalanchoe daigremontiana/pinnata* (cathedral bells)	Psychic agitation, restlessness, and anxiety	4512	200	745		
*Viscum album *(mistletoe extract)	Cancer	2251				
*Aconitum napellus *(monkshood)	(Neuropathic) Pain	1468	500			20 000
Aurum (gold)	Harmonisation of salutogenic forces	1570	2700	50		3 500
Argentum (silver)	Activation of anabolic processes	1330	90			
*Arnica montana *(arnica)	Traumatic injury	638	4210			18 820
*Fragaria vesca/Vitis vinifera *(strawberry/vine leaves)	Toxic hepatic damage				8200	

^a^Comprising plants and metals in different concentrations, partly also in combination with other ingredients.

**Table 2:** Demographic data of admitted patients and of patients suffering from ADRs^a^.

	Patients with ADR	Hospitalized patients
Number (%)	174 (4.6)	3813
Gender (*n* male/*n* female, %)	43/131 (62.1)**	1750/2063 (54.1)
Age (median, IQR)	72, 61.0; 80.0 **	67, 55.0; 77.0

^a^Multiple hospitalizations of the patients were counted as separate cases of the same patient. **P < 0.001 all hospitalized versus patients with ADR.

**Table 3:** Characterization of adverse drug reactions (ADRs) of hospital in-patients according to severity grade and seriousness.

ADR characteristics	
Total number identified	211
Severity grade CTCAE (*n*, %)	
II/III	191, 90.5%
IV/V	19, 9.5%
Serious (*n*, %)	55, 26.1%

**Table 4:** Drugs most frequently associated with the identified adverse drug reactions (ADRs)^a^.

Drug group (ATC-Code)	No (%) ADRs	Drugs (number of ADRs for each causative drug)	Adverse drug reactions main symptoms	CTCAE severity upgrade (*n*)
Opioids (N02A)	35	Morphine (23), fentanyl (4), tramadol (4), oxycodone/naloxone (1), hydromorphone (1), oxycodone (1), and piritramide (1)	Constipation (19), nausea (9), nightmares (3), itching (1), disorientation (1), seizure (1), vomiting (1), emesis (1), hyperacusis (1), sedation (1), and haemolytic anaemia (1)	Grade II (28) grade III (7)

Penicillin/beta-lactamase inhibitors (J01C)	40	Sultamicillin (17), piperacillin (14), combactam (8), and amoxicillin (1)	Diarrhoea (10), *C. difficile*-associated diarrhoea (6), nausea (3), dyspnoea (2), emesis (2) allergic reactions (2), drug eruption (1), renal failure (1), and vaginal mycosis (1)	Grade II (22) grade III (17), and grade IV (1)

Cephalosporin/ Carbapenem (J01D)	36	Cefuroxime (23), ceftriaxone (10), and imipenem/cilastatin (3)	*C. difficile*-associated diarrhoea (12), diarrhoea (6), drug eruption (6), anaphylactic reaction grade III (1), seizures (1), tongue swelling (1), angioedema (1), renal failure (1), and collapse (1)	Grade II (16), grade III (16), grade IV (3), grade V (1)

Corticosteroids for Systemic Use (H02AB)	17	Prednisolone (7), dexamethasone (4), prednisone (5), and methylprednisolone (1)	Hyperglycaemia (10), psychotic reaction (2), confusion (1), hypertension (1), cephalgia (1), secondary sepsis (1), gastric ulcer (1), and oesophagitis (1)	Grade II (2), grade III (11), grade IV (4), and grade V (1)

Platelet aggregation inhibitors excluding heparin (B01AC)	24	Clopidogrel (6) and ASA (18)	Bleeding (11), anaemia (2), abdominal pain (1), gastric ulcer (1), and gastritis (1)	Grade II (14), grade III (4), and grade IV (6)

Loop diuretics (C03CA)	16	Furosemide (10) and torasemide (6)	Renal failure acute or chronic (12), hypotension (2), exsiccosis (2), itching (1), uraemia (1), pemphigoid reaction (1), and electrolyte disorder (1)	Grade II (9), grade III (4) grade IV (1), and grade V (2)

Quinolones (J01M)	15	Levofloxacin (7), ciprofloxacin (7), and moxifloxacin (1)	*C. difficile*-associated diarrhoea (2), diarrhoea (2), and increase in GT (2), alkaline phosphatase (2), vertigo (1), seizure (1), and constipation (1)	Grade II (6) and grade III (9)

Macrolides (J01F)	13	Clarithromycin (9) and clindamycin (4)	*C. difficile*-associated diarrhoea (5), diarrhoea (3), membranous colitis (1), drug fever, (1) allergic reaction (1), and drug eruption (1)	Grade II (7) and grade III (6)

Heparin group (B01AB)	10	Enoxaparin (8) and heparin (2)	Bleeding (10)	Grade II (6), grade III (2), and grade IV (2)

Oral Antidiabetics (A10B)	11	Metformin (8) and exenatide (3)	Diarrhoea (7), meteorism (4), abdominal pain (1), nausea (1), and acute renal failure (1)	Grade II (10) and grade III (1)

^a^Multiple drugs may be attributed to one or more symptoms, as well to severity grades (CTCAE).

**Table 5:** Adverse drug reactions (ADRs) caused by complementary and alternative medicine (CAM) drugs.

Indication	Patients age, sex	Drug ingredient and route of application	Concomitant medication also possibly related* and route of application	ADR	Type of ADR	Causality
Rheumatoid arthritis	62, f	*Equisetum/Formica s.c.*	Arnica/levisticum s.c.	Local skin reaction (erythema)	B	Probable
Rheumatoid arthritis	62, f	Mistletoe plant extract^a1^ s.c.	—	Local skin reaction (erythema) (8 × 8 cm)	A	Probable
Mesothelioma cancer	65, m	Mistletoe plant extract^a2^ s.c.	—	Temp. >38.6°C; Local skin reaction (erythema) 15 × 10 cm	A	Probable
Breast cancer	41, f	Mistletoe plant extract^a3^ s.c.	Pamidronate, i.v	Temp. 39.6°C	A	Possible
Chronic hepatitis C	35, f	*Fragaria vesca/Vitis vinifera p.o. *		Maculopapular exanthema forearms	B	Possible
Depression	53, f	*Gentiana, Bryophyllum p.o.*	Metamizole p.o.	Drug eruption	B	Possible

*Drugs for which the causal relationship to the same ADR was also considered to be possible; ^a1^Iscucin salicis A; ^a2^Abnoba viscum fraxini 20 mg; ^a3^Helixor M 1 mg. s.c.: subcutaneous, p.o.: per os, i.v. and intravenous.
